# Areca Nut Extract Induces Pyknotic Necrosis in Serum-Starved Oral Cells via Increasing Reactive Oxygen Species and Inhibiting GSK3β: An Implication for Cytopathic Effects in Betel Quid Chewers

**DOI:** 10.1371/journal.pone.0063295

**Published:** 2013-05-21

**Authors:** Wen-Tsai Ji, Cheng-I Lee, Jeff Yi-Fu Chen, Ya-Ping Cheng, Sheng-Ru Yang, Jung-Hua Chen, Hau-Ren Chen

**Affiliations:** 1 Department of Life Science, Institute of Molecular Biology and Institute of Biomedical Science, College of Science, National Chung Cheng University, Min-Hsiung, Chia-Yi, Taiwan; 2 Department of Biotechnology, Kaohsiung Medical University, Kaohsiung, Taiwan; Temple University, United States of America

## Abstract

Areca nut has been proven to be correlated with various pathologic alterations in oral cavity. However, the mechanisms for such cytopathic effects are still elusive due mostly to the limitations of cell culture systems. Here we discovered that areca nut extract (ANE) induced production of autophagosome vacuoles in cells cultured with rich medium but induced pyknosis and ballooning, two morphological alterations frequently observed in betel quid chewers, in cells under a serum-free culture condition. Permeability of the serum-starved cells to propidium iodide (PI) confirmed ANE induced novel necrosis with pyknosis (pyknotic necrosis), providing a possible explanation for inflammatory infiltration in chewers’ mucosa. In these serum-starved cells, ANE strongly induced reactive oxygen species (ROS), which acted as a key switch for the initiation of pyknotic necrosis. Calcium flux was also involved in the morphological alterations. Besides, inhibition of GSK3β by SB216763 significantly exacerbated the pyknotic necrosis either induced by ANE or H_2_O_2_ in serum-starved cells, suggesting that GSK3β is a critical regulator for ANE/ROS-mediated pyknotic necrosis. Interestingly, LC3-II transition and PARP cleavage were still detected in the serum-starved cells after ANE treatment, suggesting concurrent activation of apoptotic and autophagic pathways. Finally, insulin could counteract the effect of ANE-induced pyknotic necrosis. Taken together, these data provide a platform for studying ANE-induced cytopathogenesis and the first clinical implication for several pathological alterations, such as ballooning and inflammatory infiltration, in betel quid chewers.

## Introduction

Betel quid consists of areca nut, inflorescence of *Piper betle* and slaked lime. Betel quid chewing is popular in South-east Asia and about 10 to 20% of the global populations are potential users [1]. Chewing of betel quid is associated with several pathological effects in the oral cavity, including ulcers, thickened epithelium, brownish discoloration, submucosal fibrosis, and pseudomembranous wrinkle alteration in chewer’s mucosa [2]. Histologically, ballooning, epithelial hyperplasia, massive inflammatory infiltration, basal nuclei hyperkeratosis, pyknosis and dysplasia have been observed [2], [3], [4].

Among the components of betel quid, areca nut extract (ANE) was reported to cause morphological alterations in cultured cells such as retraction and cytoplasmic vacuoles [5]. Subsequent studies confirmed that the vacuole formation was due to ANE-induced, ROS-mediated autophagy [6]. ANE also caused cell cycle arrest and senescence in oral keratinocytes [6], [7]. Besides, a few compounds in areca nut are cytotoxic to various cell lines. For example, arecoline, a major alkaloid of areca nut, is genotoxic and may contribute to oral carcinogenesis by causing DNA damage and downregulation of cyclin-dependent kinase inhibitors p21 and p27 [8]. Treatment of arecoline induces apoptosis and anoikis in basal cell carcinoma cells and HA22T/VGH cells, respectively [9], [10]. Areca nut-derived oligomericprocyanidins has also been proven to induce apoptosis in human lymphocytes [11].

Although areca nut is associated with several pathologic alterations in oral cavity, most of the cytopathic effects including ballooning and inflammatory infiltration cannot be simulated in regular culture systems. In this study, we established a culture condition for studying the ANE-induced pyknotic necrosis, which resembles more closely to the cytopathic condition *in vivo*. Moreover, we showed that ROS accumulation and GSK3β inhibition played important roles in this ANE-induced novel necrosis.

## Materials and Methods

### Cell Culture

Oral cancer cell line SAS, a gift kindly provided by Dr. Jeff Yi-Fu Chen, was maintained in DMEM supplemented with 10% fetal bovine serum (FBS) and 1% penicillin/streptomycin [12]. OCSL and OC2, two oral squamous cell carcinoma cell lines derived from two Taiwanese men with habits of drinking, smoking, and areca nut chewing, were maintained in RPMI1640 medium with similar supplements [13]. Normal human oral keratinocytes (NHOKs) were purchased from ScienCell (Carlsbad, CA, USA) and maintained in the recommended oral keratinocyte medium (OKM) with oral keratinocyte growth supplement (OKGS). Cells were routinely kept in a 37°C incubator supplied with 5% CO_2_ and subcultured every two to three days. Twelve to sixteen hours after seeding, experiments were performed soon after medium refreshing when cell confluence was about 20–50%. For serum starvation, cells were washed twice and cultured in serum-free medium immediately before treatment.

### Areca Nut Extract

Areca nut extract (ANE) was prepared as described previously [14]. The nuts were chopped into about 0.5–1 cm^3^ dices by a blender and the water-soluble ingredients were extracted at 4°C overnight. The supernatant was collected and concentrated by lyophilisation at −70°C. The lyophilized powder was weighed, re-dissolved in ddH_2_O, and stored at −20°C before use.

### Reagents and Antibodies

N-acetylcysteine (NAC), 2′–7′-Dichlorofluorescin diacetate (DCFDA), ammonium chloride (NH_4_Cl), EGTA, acridine orange (AO), propidium iodide (PI), dimethyl sulfoxide (DMSO), the calcium chelator 1,2-bis (o-aminophenoxy)- ethane-N,N,N',N'-tetraacetic acid (BAPTA), and 4′, 6-Diamidino-2-phenylindole (DAPI) were purchased from Sigma-Aldrich (St. Louis, MO, USA). Z-VAD-fmk and GSK3β inhibitor SB216763 were from Merck (Frankfurter, Germany). Antibodies against GSK3β and LC3 were from Cell Signaling Technology (Danvers, MA, USA). Antibodies of phosphorylated GSK3β (Ser9) and cleaved PARP (24 kDa) were from Epitomics (Burlingame, CA, USA). The antibody of γH2AX was from Abnova (Walnut, CA, USA).

### DAPI and Acridine Orange (AO)/ethidium Bromide (EtBr) Staining

For DAPI staining, cells were washed twice with PBS and fixed with ice-cold 4% paraformaldehyde for 20 minutes. After washing again, cells were stained with DAPI/PBS. About ten minutes later, cells were washed, kept in PBS and observed under the fluorescence microscope. For AO/EtBr staining, AO/EtBr mixture with a final concentration of 10 µg/ml was added into the medium. Ten minutes later, cells were washed, kept in PBS and observed immediately.

### Nucleus Size Evaluation

After DAPI staining and photography, the longitude of the nucleus in each cell was evaluated using the software Image-pro Express 6.0. Sizes of a total of 200 nuclei in each microscopic vision field (100X) were measured and averaged. The results from five different fields were statistically analyzed.

### DNA Fragmentation Analysis

DNA fragmentation assay was processed as described previously [15]. In brief, cells in 10 cm plates were scraped into culture medium and harvested by low-speed centrifugation 48 hours after ANE treatment. After washing twice with PBS, cells were lysed in a buffer composed of 10 mM Tris-HCl (pH 7.5), 10 mM EDTA, and 1% sodium dodecyl sulfate (SDS). After addition of equal amount of 2 M NaCl, the extracts were incubated for 12 hours at 4°C. Subsequently, the lysates were centrifuged for 10 min at 10,000 × *g* and the derived supernatant was transferred to new tubes. Final doses of 200 µg proteinase K/ml and 50 µg RNase A/ml were added into the mixture. After incubation for 1 h at 37°C, DNA was harvested by phenol-chloroform extraction and 95% ethanol precipitation. The pellet was redissolved in TE buffer containing 50 µg RNase A/ml and run by electrophoresis in 1.5% agarose gels.

### ROS Detection

ROS was quantified as previously described [8]. Cultured cells in 24 wells were pretreated with 10 µM DCFDA for 30 minutes. Then, cells were washed twice with serum free or normal medium and incubated continuously. At indicated time points after ANE treatment, cells were finally washed twice with PBS and dissolved in 200 µl DMSO containing 1 mM NAC for quenching reaction. After swirling for seconds, 50 µl of supernatant was transferred for fluorescence evaluation.

### Quantification of Intracellular Calcium

Cells cultured in 35 mm dish were washed twice with PBS and continuously incubated in fresh FBS-supplemented medium containing 2.5 µM Fluo-4 acetoxymethyl ester (Fluo-4/AM) (Molecular Probe) for 1 hour. Then, cells were washed thrice and further cultured in Hank’s buffer saline for 30 minutes to allow complete removal of the ester group of the calcium indicator. After treatment with ANE or thapsigargin (TG), cells were continuously photographed thrice at each time point with 2 seconds exposure time under 450–490 nm excitation. The integrated optical density per area of cells in certain microscopic vision fields was obtained using the software Image-pro 3DS 5.1. The relative intensity was obtained by comparing the intensity of experimental results with that of time zero which was deliberately set to 1. Meanwhile, the photos were colored by Image-pro Express 6.0.

### Dual Staining of Propidium Iodide (PI) and Annexin V

After indicated treatment, cells were washed twice with PBS and dually stained with PI and annexin V-FITC using Annexin V-FITC detection kit (Strong Biotech) according to the manufacturer’s instruction. The result was observed under the fluorescence microscope and photographed.

### Cell Lysate Preparation and Western Blot

Cell lysate preparation and Western blot were performed as described [8].

### Statistical Analysis

All data were analyzed using Student’s t-test and the results with p value smaller than 0.05 were defined to be significant.

## Results

### Areca Nuts Extract Induced Pyknotic Necrosis in Serum-starved Oral Cells

**Figure 1:**
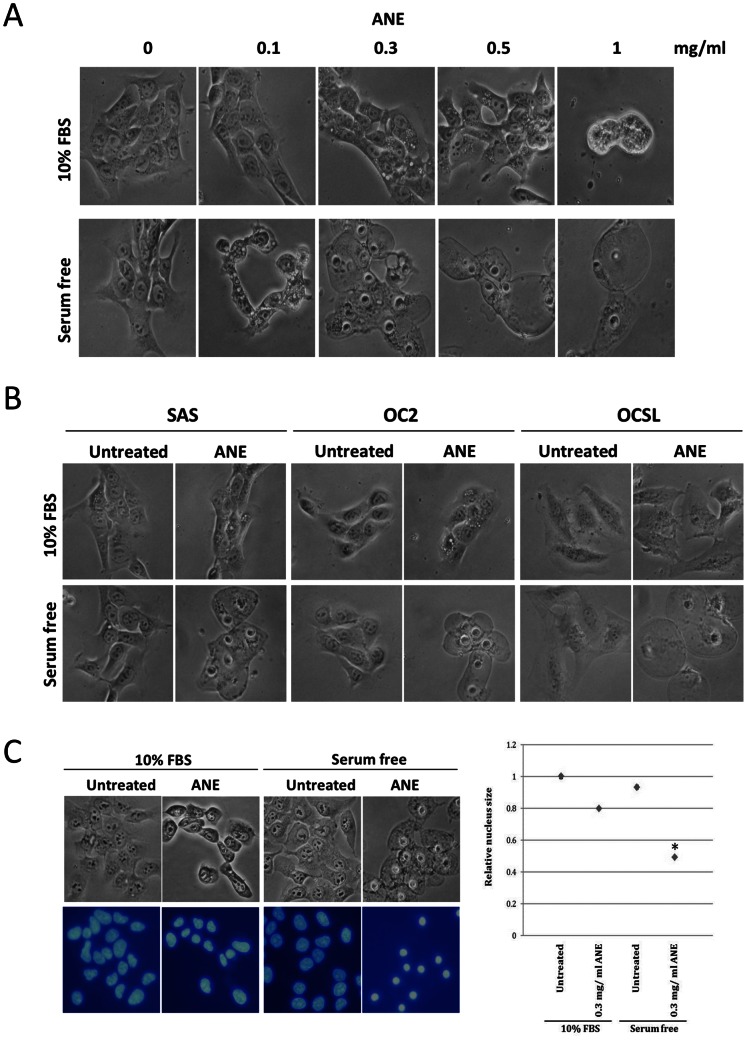
ANE-induced pyknosis and cell ballooning in serum-starved oral cells. (A) SAS cells mock-treated or treated with various doses of ANE were cultured in medium supplemented with or without 10% FBS for 6 hours and photographed. (B) Similar to the condition in (A), SAS, OC2 and OCSL with 0.5 mg/ml ANE treatment were observed. (C) SAS cells were treated with 0.3 mg/ml ANE for 12 hours, fixed and stained with DAPI. Nuclei size was evaluated as described in experimental procedures. The average size of nuclei of untreated FBS-supplemented cells was defined as 1.

Although many studies have shown significant correlation between areca nut and various oral pathologic alterations, the mechanisms of areca nut-induced effects have been elusive. Consistent with previous reports, 1 mg/ml ANE induced autophagic vacuoles and retraction within 6 hours in FBS-supplemented SAS cells (Fig. 1A upper panel). In contrast, ANE treatment resulted in nucleus shrinkage (pyknosis) and ballooning in serum-starved SAS cells, strongly suggesting that ANE may cause deregulation of water permeability or necrosis under serum-free conditions. These effects might be universal because similar results were observed in two other cell lines, OC2 and OCSL, as well as in normal human oral keratinocytes (NHOKs) (Figs. 1B, S1). DAPI staining further confirmed the pyknosis in ANE-treated/serum-starved cells, although the average nuclear size was also slightly reduced in ANE-treated/FBS-supplemented cells (Fig. 1C).

**Figure 2:**
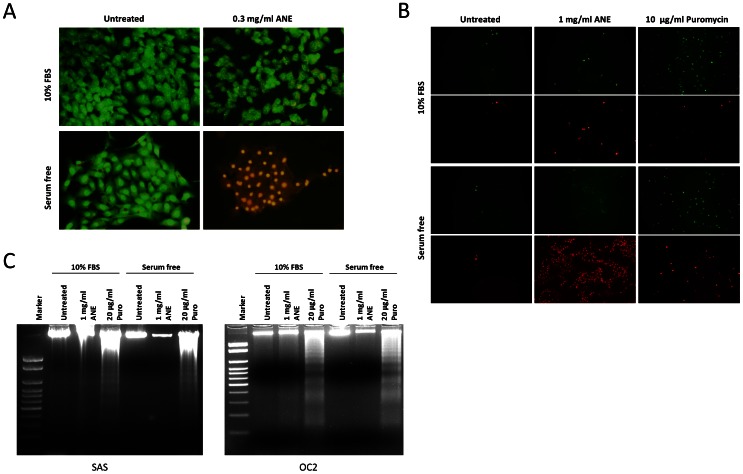
Necrosis was induced by ANE in serum-starved cells. (A) SAS cells were treated with 0.3 mg/ml for 12 hours and stained with acridine orange (AO) and ethidium bromide (EtBr). (B) SAS cells treated with 1 mg/ml ANE for 24 hours were stained with Annexin V (green) and propidium iodide (red). Cells treated with 10 µg/ml puromycin were used as the positive control of apoptosis. (C) SAS and OC2 cells treated with 1 mg/ml ANE or 20 µg/ml puromycin were harvested for DNA fragmentation analysis 48 hours after treatment.

To verify whether ANE caused necrosis, cells were dually stained with acridine orange (AO) and the membrane-impermeable reagent ethidium bromide (EtBr). As shown in Fig. 2A, only the ANE-treated/serum-starved cells were stained by EtBr (Fig. 2A). Cells treated with puromycin, a proven reagent that induces apoptosis, were more prone to annexin V staining than to propidium iodide (PI) [16]. However, in contrast to puromycin-treated/serum-starved cells, ANE-treated/serum-starved cells only showed weak staining of annexin V but strong staining of PI (Fig. 2B). In addition, ANE did not cause DNA ladder formation under serum-free conditions (Fig. 2C). Taken together, these results suggest that ANE induced pyknotic necrosis rather than the canonical apoptosis in serum-starved cells.

### Areca Nut Extract Induced Necrosis via the Increase of Reactive Oxygen Species (ROS)

**Figure 3:**
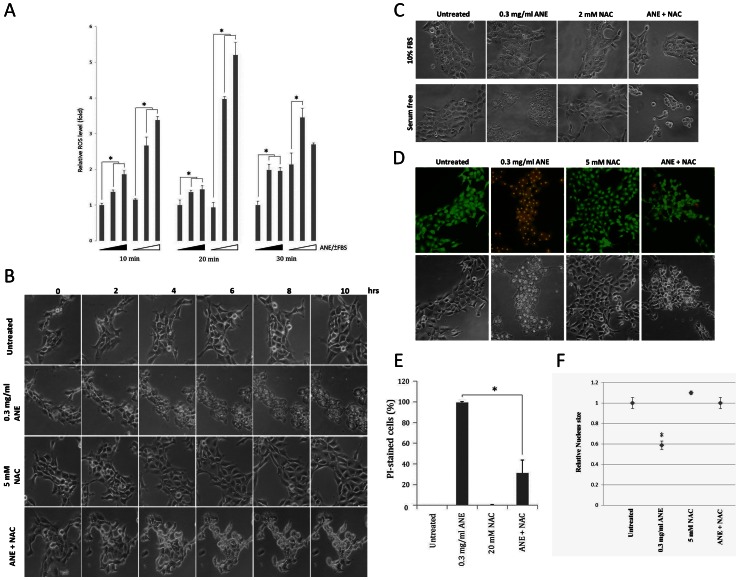
ANE-induced pyknotic necrosis via the increase of ROS. (A) ROS in SAS cells mock-treated or treated with 0.5 or 1 mg/ml ANE for different time periods was quantified. Solid triangle represented serum supplementation. The ROS level without ANE treatment was considered as 1. (B) SAS cells treated with 0.3 mg/ml ANE in the presence or absence of indicated doses of NAC were monitored at the indicated time points. (C) Similar to the condition in (B), cells in serum-supplemented or -free condition were photographed 12 hours later. (D) Similar to the condition in (C), cells were stained with AO/EtBr. Alternatively, cells were stained with PI 6 hours later (E). The percentage of stained cells was quantified. (F) The average size of nuclei of the treated cells was evaluated.

Triggered by various stimuli, ROS is well known as a potential inducer of necrosis [17], [18], and ANE has been reported to induce ROS in FBS-supplemented cells [5], [19]. However, higher levels of ROS were detected in serum-starved than those in FBS-supplemented cells (Fig. 3A). Under serum-free conditions, ANE increased autophagic vacuoles initially, finally resulting in pyknosis and ballooning (Fig. 3B). However, co-treatment of N-acetylcysteine (NAC), a ROS scavenger, completely abolished ANE-induced pyknosis and ballooning (Figs. 3B, 3C). After ROS quenching, cell morphology was rescued close to that under FBS-supplemented conditions. Consistently, co-treatment of NAC also significantly reduced the percentage of EtBr- or PI-stained cells and reversed the nucleus size (Figs. 3D–F, S2). All these results highly suggest that ANE induced pyknotic necrosis through a ROS-dependent mechanism.

**Figure 4:**
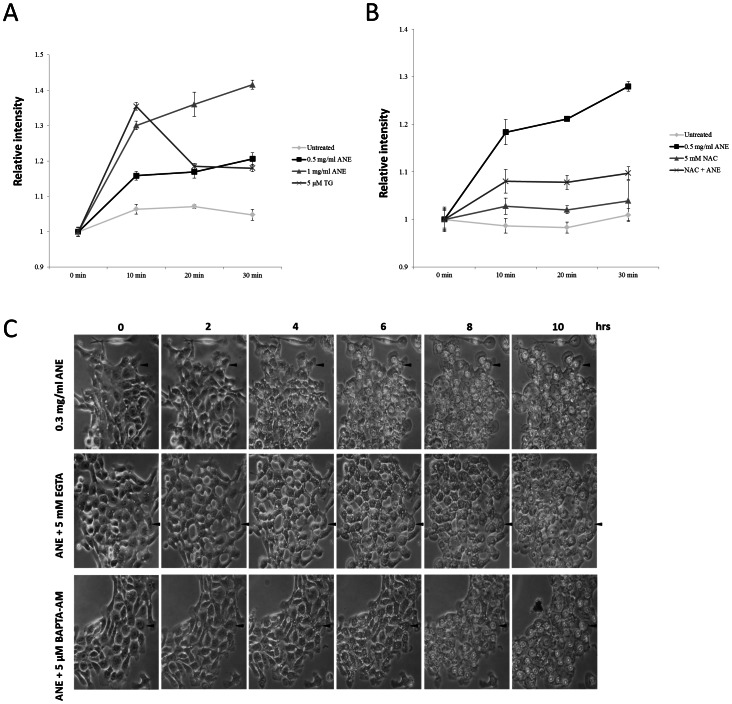
Calcium signaling was involved in ANE-induced pyknotic necrosis. (A) Calcium flux in SAS cells treated with the indicated doses of ANE was measured as described in experimental procedures. (B) Similar to the condition in (A), calcium flux was evaluated in the presence or absence of NAC. (C) Cells co-treated with ANE and either 5 mM EGTA or 5 µM BAPTA-AM were photographed at the indicated time points.

Since several studies have demonstrated that calcium is an important mediator in ROS-related necrosis, the involvement of calcium in ANE-induced necrosis was also examined [17], [18]. Indeed, ANE induced calcium flux, which decreased obviously in the presence of NAC (Figs. 4A, 4B, S3). Chelating of extracellular calcium by EGTA and intracellular calcium by BAPTA delayed pyknosis for around four and two hours respectively (Fig. 4C), suggesting that calcium also played a role in ANE-induced pyknotic necrosis.

### Inactivation of GSK3β Enhanced Pyknotic Necrosis

Although ANE caused pyknotic necrosis in serum-starved cells through a ROS-dependent mechanism, serum starvation alone increased ROS with time but was not sufficient to cause necrosis (Fig. 3A). These results suggest the possibility that other factor(s) than ROS might contribute to the ANE-induced necrosis under serum-free conditions.

**Figure 5:**
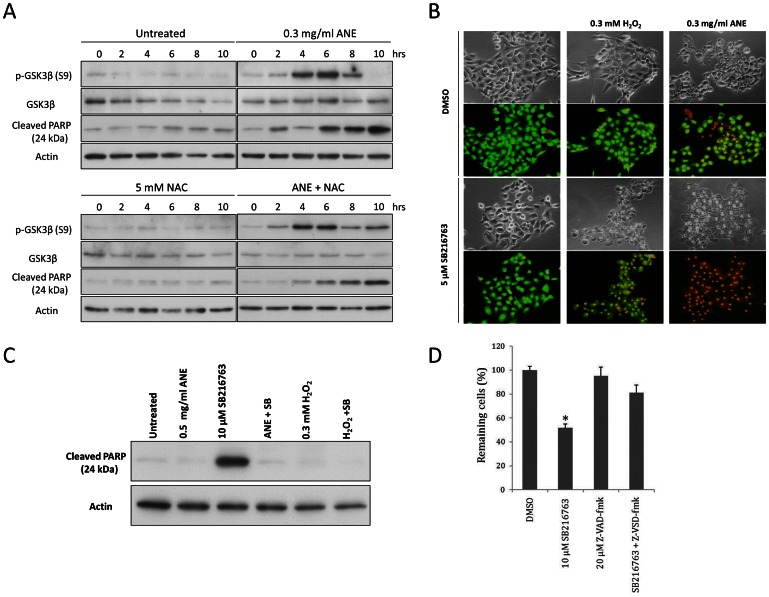
Inactivation of GSK3β enhanced pyknotic necrosis. (A) Serum-starved SAS cells treated with 0.3 mg/ml ANE and with or without 5 mM NAC were harvested at the indicated time points for detecting GSK3β, p-GSK3β and cleaved PARP by Western blot. (B) Serum-starved cells treated with SB216763 in the presence of H_2_O_2_ or ANE were stained with AO/EtBr. (C) Similar to the condition in (B), cells were harvested for Western blot analysis 4 hours after treatment. (D) Viability of serum-starved cells treated with SB216763 in the presence or absence of 20 µM Z-VAD-fmk was measured using MTT assay 16 hours after treatment.

We further identified the factor by testing various kinase inhibitors, and discovered the involvement of GSK3β in ANE-induced pyknotic necrosis under serum-free conditions. As shown in Fig. 5A (upper panel), ANE enhanced phosphorylation of GSK3β, with the peak induction 6 hours after the treatment, suppressing its activity in serum-starved cells. Co-treatment of NAC had little effect on the induction of GSK3β phosphorylation, but slightly prolonged the duration of GSK3β phosphorylation (Fig. 5A, lower panel), implying that at least the initiation of GSK3β inactivation by ANE is ROS-independent. Moreover, under serum-free conditions, ANE treatment combined with inhibition of GSK3β by SB216763 significantly exacerbated the pyknotic necrosis and rendered most of the cells susceptible to EtBr staining within 4 hours (Fig. 5B). In fact, obvious pyknosis and ballooning could be observed even as early as 2 hours after ANE treatments (data not shown). Co-treatment of GSK3β inhibitor and ANE (or H_2_O_2_) significantly countered the induction of poly-ADP ribose polymerase (PARP) cleavage by GSK3β inhibition, suggesting that inhibition of GSK3β has a major effect in enhancing necrosis rather than activating caspases under increased ROS condition (Figs. 5C, 5D).

### Areca Nut Extract Caused Pyknotic Necrosis Despite Activation of Caspases and Autophagy Cascades

**Figure 6:**
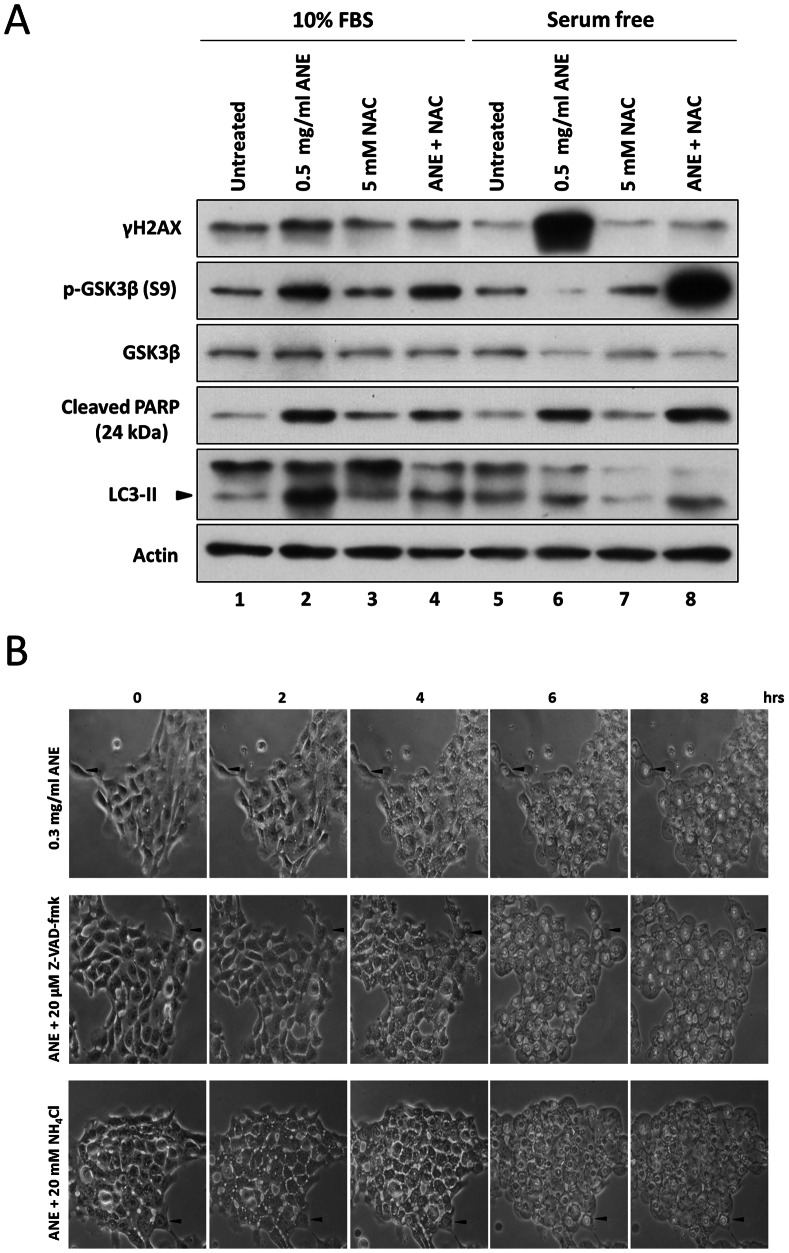
Inductions of caspase activation/autophagy and ANE-induced pyknotic necrosis were not mutually exclusive. (A) SAS cells treated with 0.5 mg/ml ANE in combination with or without 5 mM NAC were harvested for Western blot analysis 12 hours after treatment. (B) SAS cells treated with 0.3 mg/ml ANE in combination with Z-VAD-fmk or NH_4_Cl were photographed at the indicated time points under serum-free conditions. Arrows indicate the reference sites for each field.

Surprisingly, we noticed that caspase 3-mediated PARP cleavage continuously increased even after ANE–induced necrosis was initiated (Figs. 5A, S4). Co-treatment of NAC did not significantly influence PARP cleavage (Fig. 5A, lower panel), indicating that ANE induced caspases activation mainly via a ROS-independent pathway. We further investigated the effects of ANE on PARP cleavage and LC3 transition in the presence or absence of serum. As expected, 0.5 mg/ml ANE increased PARP cleavage and LC3-II in cells cultured in rich and serum-free media (Fig. 6A lane 1 vs. lane 2 and lane 5 vs. lane 6). However, the PARP cleavage and LC3-II transition were not affected by NAC co-treatment in serum-starved cells (Fig. 6A lane 6 vs. lane 8). Furthermore, neither caspases inhibition by z-VAD-fmk nor autophagy blockage by NH_4_Cl had obvious effects on the dynamic changes of pyknosis and cell ballooning (Fig. 6B). However, ANE caused severe DNA damage under serum-free conditions by a ROS-dependent mechanism, as evidenced by the dramatic change of γH2AX level (Fig. 6A, lane 6 vs. lane 8). Therefore, ANE caused pyknotic necrosis despite concurrent activation of caspases and autophagy cascades.

### Insulin Counteracted Areca Nut Extract –induced Pyknotic Necrosis

**Figure 7:**
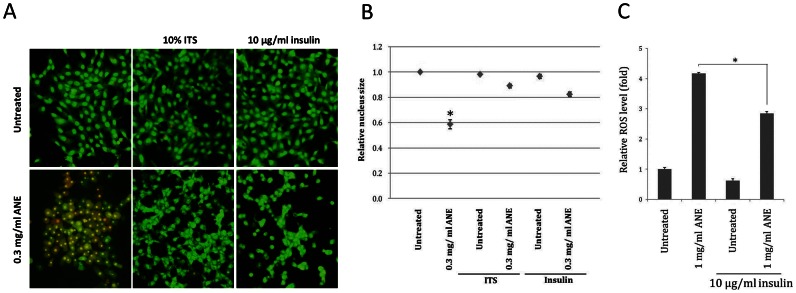
Insulin can counteract the effect of ANE-induced pyknotic necrosis. Serum-starved SAS cells were treated with ANE in combination with ITS or with insulin. Cells were analyzed by AO/EtBr staining (A). The nucleus size was assessed by DAPI staining (B), and the relative ROS level (C) was measured as mentioned above.

Our data have demonstrated that ANE induced pyknotic necrosis only under serum-free conditions, suggesting that certain components in the serum may counteract the effect of ANE. After testing the mixture of insulin-transferrin-selenium (ITS), an alternative supplement for cell culture, we discovered that insulin could counteract ANE-induced pyknotic necrosis. Under serum-free conditions, addition of either insulin/transferrin/selenium (ITS) or insulin alone was sufficient to reduce the percentage of EtBr-stained cells or to rescue nucleus size after ANE treatment (Figs. 7A, 7B, S5). Interestingly, the ANE-induced morphological alteration was similar between FBS-supplemented and ITS/insulin-treated cells (Figs. 7A, S5). In addition, treatment of ITS or insulin also significantly minimized ANE-induced ROS under serum-free conditions (Fig. 7C). These results suggest that insulin might be a potential component in serum that counteracts ANE-induced necrosis.

## Discussion

Areca nut extract (ANE) has been reported to induce autophagy and cell retraction in several studies (6, 7). Unexpectedly, we discovered that ANE caused novel morphological alterations in serum-starved oral cells. In this study, we confirmed ANE-induced ballooning and pyknotic necrosis under serum-free conditions. These results provide the cytopathic implication for betel quid chewers despite the fact that clinical pyknosis is more frequently associated with basal cells because superior squamous cells have smaller nuclei [20]. Similar effects could be observed in cells incubated in phosphate buffered saline (PBS) (data not shown), indicating that ANE could induce pyknosis in nutrient-deprived cells. Medium or PBS containing FBS with concentrations lower than 1% was still insufficient to completely reverse the morphological alterations (Fig. S6). We envision that this *in vitro* model of ANE-induced pyknosis should be recapitulated *in vivo* since oral epithelial cells have a propensity to reside in a nutrient-limited environment. However, ANE also slightly but irregularly reduced nucleus size in FBS-supplemented cells possibly due to cell retraction or apoptosis induction. ANE-induced inhibition of mTOR complex 1, which has been reported to regulate cell size, might also contribute to the retraction [8], [21].

In our results, ANE induced pyknotic necrosis via a ROS-dependent mechanism. Induction of necrosis might explain why infiltration of inflammatory cells is frequently observed in chewer’s oral mucosa [2]. Unlike ANE, 0.3 mM of H_2_O_2_ could induce necrosis even under serum-supplemented conditions at the later stage of the treatment (data not shown). However_,_ ANE induced necrosis only under serum-free conditions. ROS contains various free radicals that may exert distinct effects on downstream signaling [22]. Thus, one possible explanation for such discrepancy is that ANE may induce different ROS species from H_2_O_2._ This can be further supported by the different necrosis pattern induced by ANE compared to that by H_2_O_2_ (Fig. S7).

**Figure 8:**
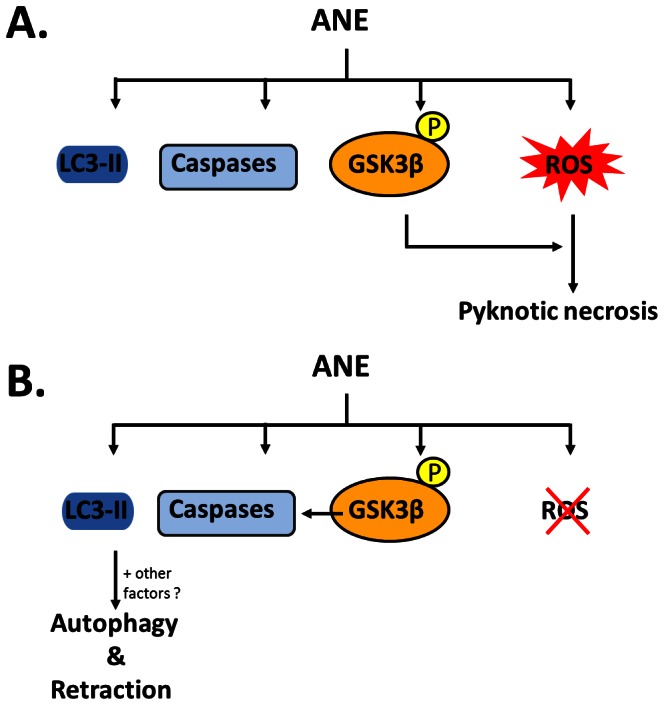
A proposed model concerning the effect of ROS and GSK3β inactivation on ANE-induced pyknotic necrosis.

Inhibition of GSK3β had been proven to be another factor by which ANE efficiently enhanced pyknotic necrosis under serum-free conditions. It has been reported that GSK3β inactivation could determine the cell fate of either apoptosis or necrosis depending on whether autophagy is present [23]. Since several studies have demonstrated that autophagy could enhance necrosis and ANE caused autophagy through ROS in oral cells, it is plausible that ANE may induce necrosis via enhancing autophagy [6], [24]. Although ANE indeed induced autophagy in our experiments (Figs. 6A, S8A), the autophagic process was not complete as evaluated by the expression level of p62, a marker of autophagy-defective cells (Fig. S8B) [25]. Moreover, inhibition of autophagy maturation by ammonium chloride (NH_4_Cl) or chloroquine did not alter the pyknotic necrosis process (Fig. 6B; data not shown). In addition, quenching ROS by NAC did not significantly reduce LC3-II and cytoplasmic vacuoles in serum-starved cells after ANE treatment (Figs. 3B, 6A). Taken together, these results disfavored the involvement of autophagy in ANE-induced pyknotic necrosis. Instead, we discovered that ROS was a novel factor in switching the roles of phosphorylated GSK3β. GSK3β inhibition alone slightly caused apoptosis in serum-starved cells but enhanced blistering and pyknotic necrosis when H_2_O_2_ was present(Figs. 5C, S7). However, in the presence of ROS, triggered either by ANE or by H_2_O_2_, PARP cleavage obviously decreased (Fig. 5C, lane 4 and lane 6), implying that ROS is a critical factor in switching the role of GSK3β inactivation from promoting apoptosis to pyknotic necrosis (Fig. 8A).

Interestingly, ANE also induced PARP cleavage at the later stage of the ongoing necrosis (Figs. 5A, 6A). These results suggest that caspase activation and pyknotic necrosis are not mutually exclusive and proceed in parallel. Such regulation of the two signaling pathways may explain why inhibition of ANE-induced necrosis by quenching ROS resulted in cell morphology similar to serum-supplemented cells (Fig. 8B). Alternatively, parallel induction of caspase activation and necrosis may be due to the integration of signals originally triggered by different components of ANE. However, in our condition, neither membrane bleb nor DNA ladder was noticed in ANE-treated/serum-supplemented cells (Figs. 1 and. 2C). Inhibition of caspases by pan-inhibitor Z-VAD-fmk also showed little effect on ANE-induced cell death and morphological alteration in FBS-supplemented cells (Fig. S9, data not shown). These results suggest that ANE can induce noncanonical or incomplete apoptosis since DNA fragmentation may be independent of apoptosis [26].

Finally, we attempted to find certain components in FBS involved in counteracting the pyknotic necrosis. In our results, insulin with high concentration alone was sufficient to counteract the effect induced by ANE. The mechanism whereby insulin antagonizes ANE effect remains unclear. Although insulin has been reported to increase cell metabolism and ROS via upregulating PI3K-Akt signaling, treatment of insulin significantly decreased ANE-induced ROS in serum-free conditions (Fig. 7C). This result suggests that insulin may minimize oxidation stress required for the subsequent necrosis. Taken together, our results successfully provide a model for studying the cytopathic effects of ANE in cultured oral cells, and a possible explanation for several clinicopathological alterations.

## Supporting Information

Figure S1 **ANE-induced pyknosis and cell ballooning in serum-starved human normal oral keratinocytes (NHOKs).**
(TIF)

Figure S2 **Effect of NAC on ANE-induced pyknosis in serum-starved SAS cells 12 hours after treatment.**
(TIF)

Figure S3 **Increase of calcium flux by different doses of ANE.**
(TIF)

Figure S4 **Inhibition of PARP cleavage by Z-VAD-fmk in serum-starved SAS cells.**
(TIF)

Figure S5 **Effects of insulin and insulin/transferrin/selenium (ITS) on ANE-induced pyknotic necrosis in serum-starved SAS cells 6 hours after treatment.**
(TIF)

Figure S6 **The effects of different FBS concentrations on the alleviation of ANE-induced pyknotic necrosis in OC2 cells.**
(TIF)

Figure S7 **Effect of H_2_O_2_ on the cell morphology alteration induced by SB216763 in serum-starved SAS cells 16 hours after treatment.**
(TIF)

Figure S8 **Induction of incomplete autophagy by ANE as revealed from the LC3-GFP puncta (A), type II LC3 accumulation (B) and p62 increase (C) within 24 hours.**
(TIF)

Figure S9 **Effect of Z-VAD-fmk on ANE-induced cell death in FBS-supplemented SAS cells 16 hours after treatment.**
(TIF)
