# Supplementary figures and images for: Areca Nut Extract Induces Pyknotic Necrosis in Serum-Starved Oral Cells via Increasing Reactive Oxygen Species and Inhibiting GSK3β: An Implication for Cytopathic Effects in Betel Quid Chewers
Source: PLoS One. 2013 May 21;8(5):e63295. doi: 10.1371/journal.pone.0063295 (PMC3660451; doi:10.1371/journal.pone.0063295)

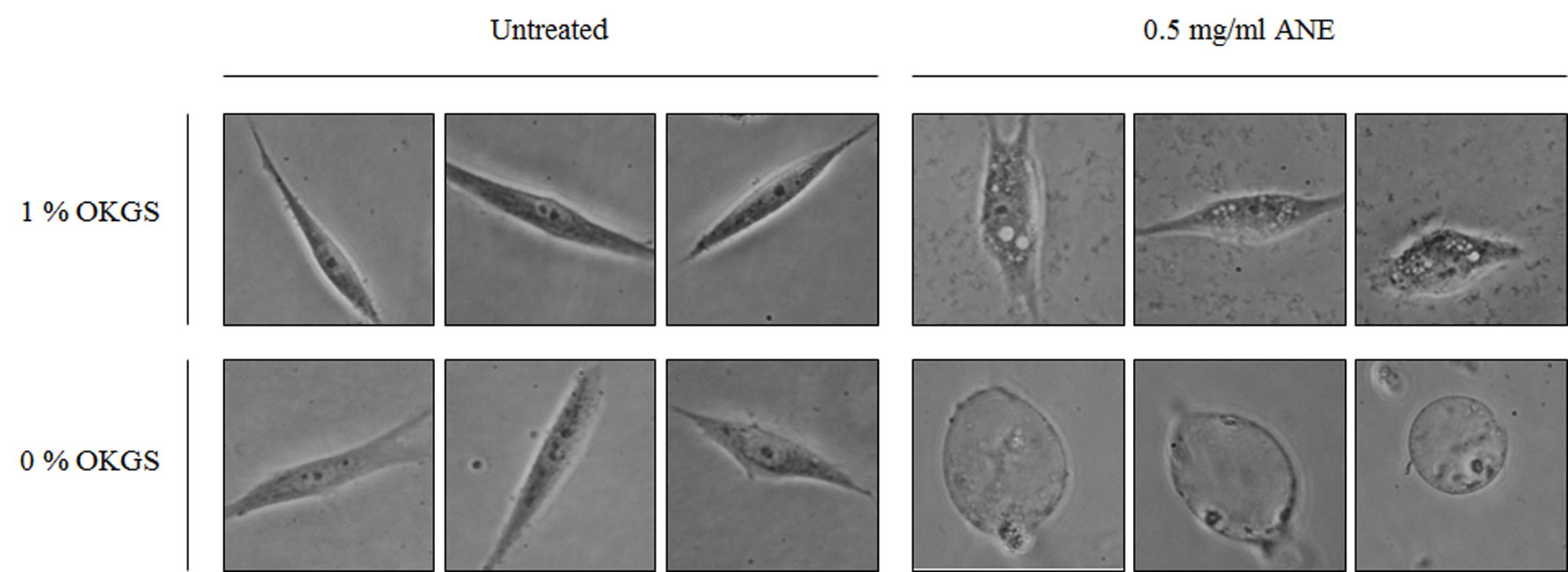

Supplement: Figure S1 — ANE-induced pyknosis and cell ballooning in serum-starved human normal oral keratinocytes (NHOKs). (TIF) [file pone.0063295.s001.tif]

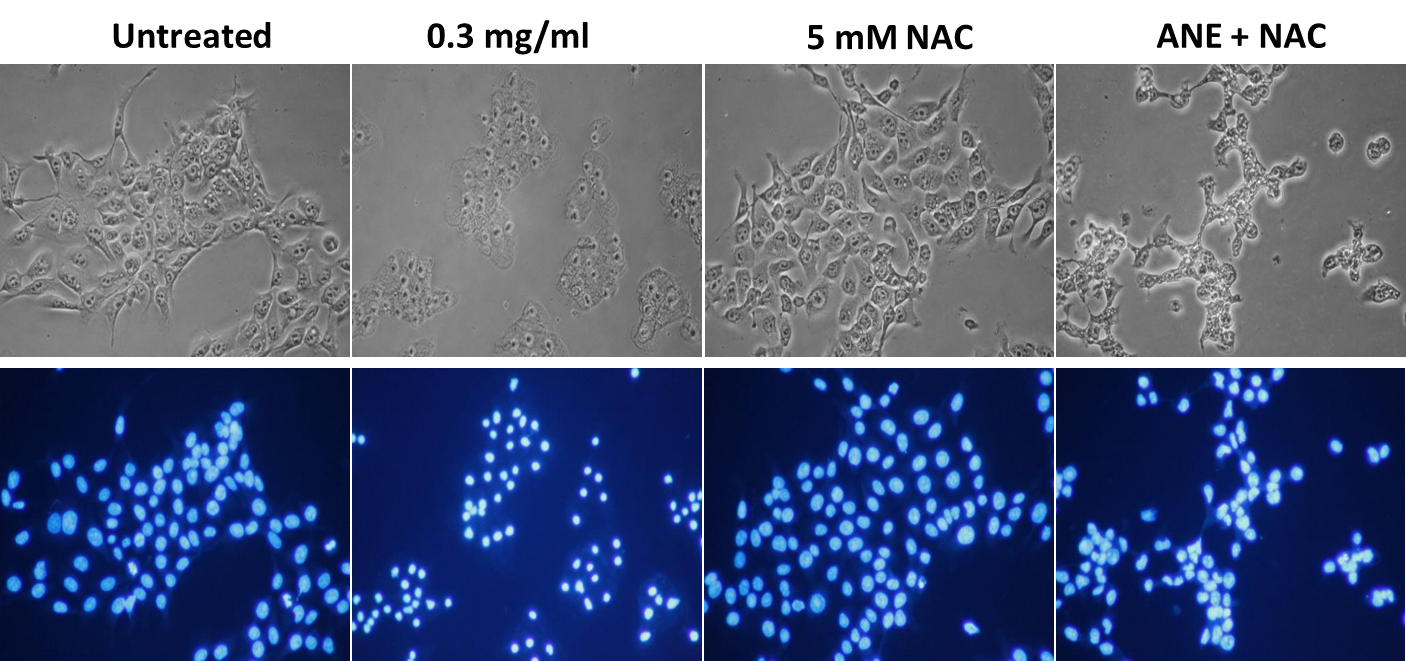

Supplement: Figure S2 — Effect of NAC on ANE-induced pyknosis in serum-starved SAS cells 12 hours after treatment. (TIF) [file pone.0063295.s002.tif]

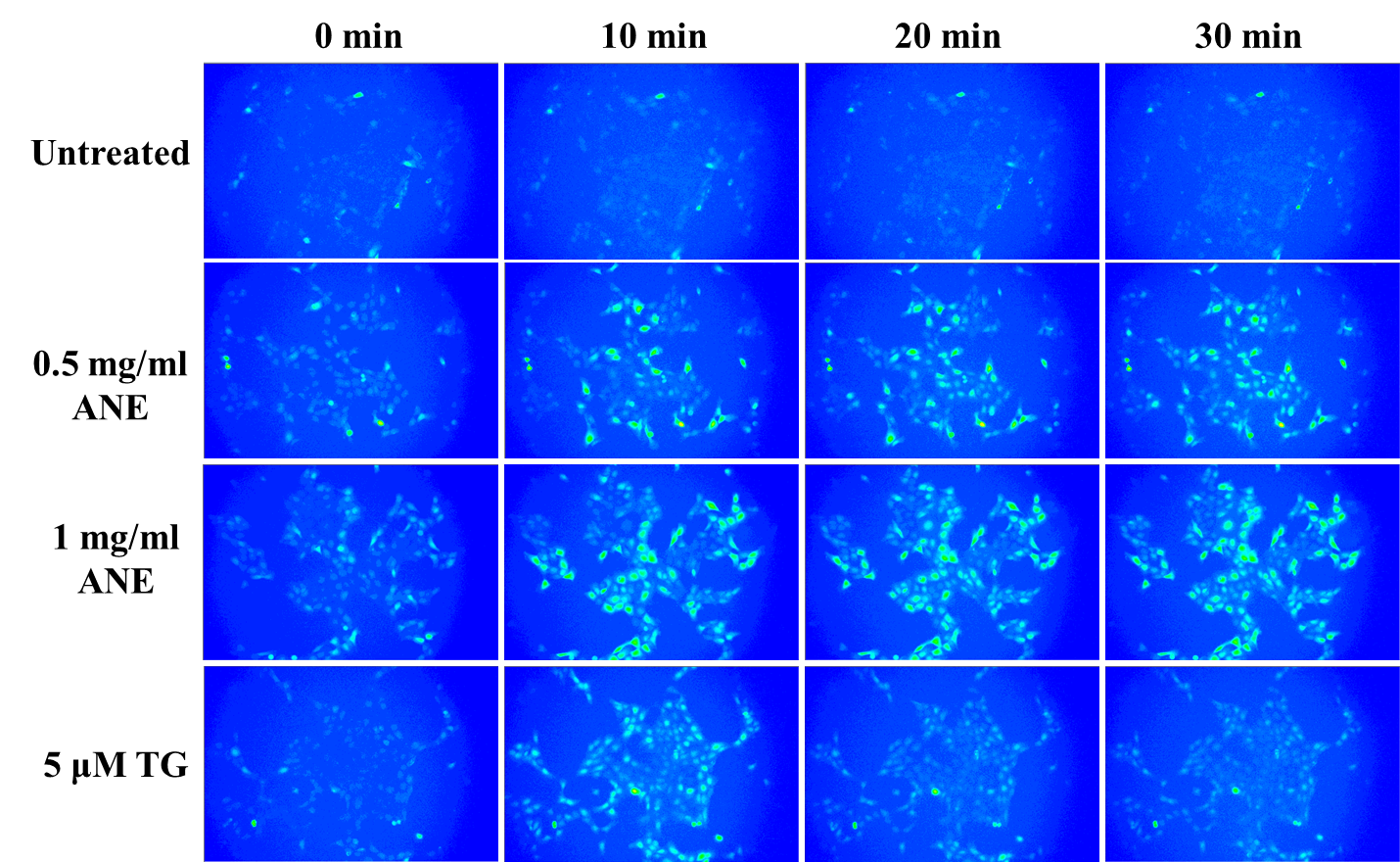

Supplement: Figure S3 — Increase of calcium flux by different doses of ANE. (TIF) [file pone.0063295.s003.tif]

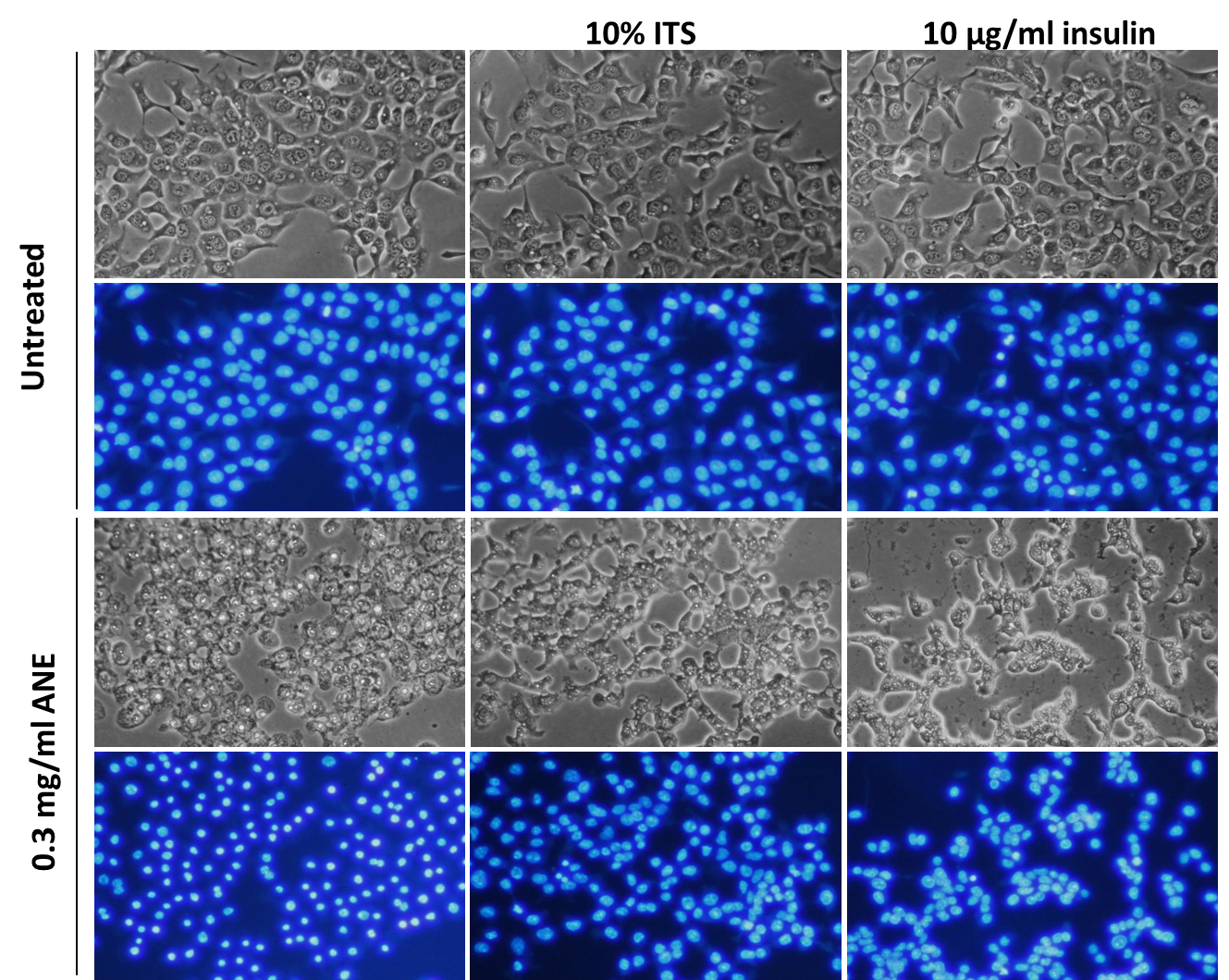

Supplement: Figure S5 — Effects of insulin and insulin/transferrin/selenium (ITS) on ANE-induced pyknotic necrosis in serum-starved SAS cells 6 hours after treatment. (TIF) [file pone.0063295.s005.tif]

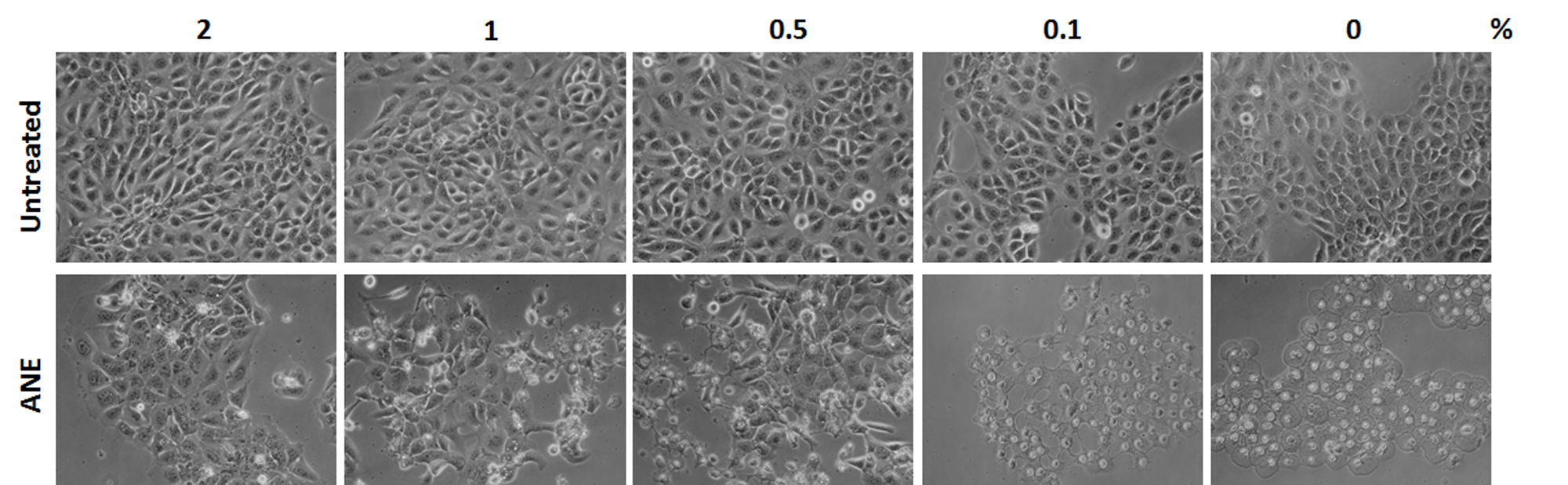

Supplement: Figure S6 — The effects of different FBS concentrations on the alleviation of ANE-induced pyknotic necrosis in OC2 cells. (TIF) [file pone.0063295.s006.tif]

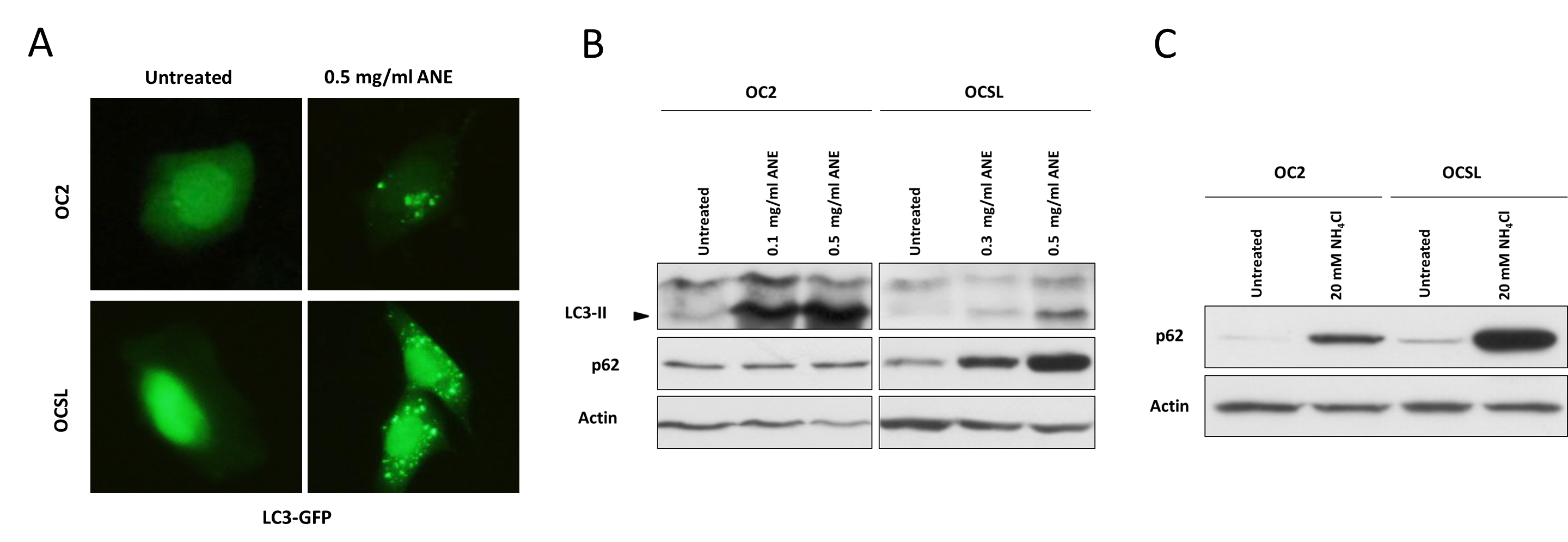

Supplement: Figure S8 — Induction of incomplete autophagy by ANE as revealed from the LC3-GFP puncta (A), type II LC3 accumulation (B) and p62 increase (C) within 24 hours. (TIF) [file pone.0063295.s008.tif]
